# Lateralized differences in power spectra across different frequency bands during NREM sleep in patients with primary insomnia

**DOI:** 10.3389/fnins.2025.1532011

**Published:** 2025-01-21

**Authors:** Jiao Huang, Jing Ye, Mingjie Gao, Wentao Gao, Weijia Chen, Yifeng Zhu, Yongbo Wang, Daijin Huang, Yunhui Lv, Hong Shi

**Affiliations:** ^1^State Key Laboratory of Primate Biomedical Research, Institute of Primate Translational Medicine, Kunming University of Science and Technology, Kunming, China; ^2^Department of Neurosurgery, The First People's Hospital of Yunnan Province, The Affiliated Hospital of Kunming University of Science and Technology, Kunming, China; ^3^Department of Sleep Center, the First People's Hospital of Yunnan Province, The Affiliated Hospital of Kunming University of Science and Technology, Kunming, China; ^4^Institute of Information Science and Engineering, Yunnan University, Kunming, China; ^5^Department of PET/CT Center, the First People's Hospital of Yunnan Province, The Affiliated Hospital of Kunming University of Science and Technology, Kunming, China; ^6^Department of Clinical Laboratory, the First People's Hospital of Yunnan Province, The Affiliated Hospital of Kunming University of Science and Technology, Kunming, China

**Keywords:** NREM (non REM) sleep, primary insomnia (PI), power spectral analysis, brain lateralization, memory consolidation, hyperarousal state

## Abstract

**Objective:**

To compare the electroencephalogram power spectrum of patients with primary insomnia and good sleep controls in multiple brain areas and different frequency bands during non-rapid eye movement sleep.

**Methods:**

48 primary insomnias and 30 age-and gender-matched good sleep controls were recorded overnight with polysomnography. Power spectral analysis was performed in six brain areas (F3, F4, C3, C4, O1 and O2) and across seven frequency bands (delta, sigma, alpha, theta, beta1, beta2, and gamma) during non-rapid eye movement sleep between primary insomnias and good sleep controls.

**Results:**

In primary insomnias, there were significant differences in frequency bands and areas. Compared to good sleep controls, delta power was lower in primary insomnias, while beta1, beta2, and gamma were higher. Beta2 power was substantially higher in all areas, sigma power was significantly higher on the right side, and gamma power was considerably higher on the left side in primary insomnias. The Beta1 power was positively correlated the number of awakenings (*r* = 0.3291, *p* = 0.02) in primary insomnias on the right side.

**Conclusion:**

This study marked the first specialized comparison of power spectral analysis during non-rapid eye movement sleep in different areas and across different frequency bands. The result suggested that primary insomnias had reduced deep sleep (lower delta power) and hyperarousal state (higher beta 2 power). Primary insomnia was associated with significant fragmented sleep, and an increase in beta1 power was related to the number of awakenings.

**Significance:**

These findings revealed the hemispheric lateralization characteristics of power spectral disturbances during non-rapid eye movement sleep in primary insomnias and provided valuable insights for selecting electrode placements in future power spectral analyses of primary insomnias.

## 1 Introduction

With the intensification of societal specialization and competition, the prevalence of insomnia has been steadily increasing, emerging as a significant health concern (Wittchen et al., 2011). Insomnia is typically diagnosed based on subjective clinical interviews, which exhibit a high degree of heterogeneity, leading to suboptimal clinical treatment outcomes (American Psychiatric Association, D, 2013; Perlis et al., 2022). The biological mechanisms of insomnia remain poorly understood. Polysomnography (PSG), the objective gold standard for sleep staging in humans and mammals (Rundo and Downey III, 2019), only affords a relatively coarse sleep quality measurement. PSG scores vary widely between different nights (Hu et al., 2024), limiting its clinical utility and the resolution of scientific inquiries. The advent and progression of quantitative electroencephalography (qEEG) offer a fresh perspective on understanding insomnia, wherein meticulous and stable power spectral analysis unveils the biological mechanisms underlying various sleep stages in insomnia (Israel et al., 2012).

The qEEG studies demonstrated increased high-frequency electroencephalography (EEG) activity during the non-rapid eye movement (NREM) sleep in insomnia (Zhao et al., 2021), suggesting that cortical hyper-excitability and hyperarousal state are involved in the brain mechanisms of insomnia (van der Werf et al., 2010; Van Someren, 2021). These high-frequency EEG activities were mostly confined to the beta band (13-30 Hz; Merica et al., 1998; Spiegelhalder et al., 2012). However, some studies proposed that high-frequency EEG activities indicative of hyperarousal state should extend into the low gamma band (30-50 Hz; Perlis et al., 2001a; Perlis et al., 2001b). Beta and gamma oscillations are more prominent during wakefulness and REM sleep (Brown et al., 2012), but enhanced gamma activity also occurs in the upstate of slow oscillations. Gamma oscillations during NREM sleep hold biological significance, such as the consolidation of declarative memories (Mena-Segovia et al., 2008; Valderrama et al., 2012). To comprehensively elucidate the biological mechanisms underlying insomnia, it is essential to independently investigate the elevated gamma and beta power observed during NREM sleep, as each may reflect distinct underlying processes contributing to the disorder.

Human memory exhibits a typical pattern of hemispheric functional specialization (Güntürkün and Ocklenburg, 2017). The declarative memory was processed mainly in the left hemisphere (Gerrits et al., 2020; Güntürkün et al., 2020). Synchronized neural activity in the gamma frequency range plays a functional role for the formation of declarative long-term memories in humans (Axmacher et al., 2006; Osipova et al., 2006).There had abundant research on hemispheric lateralization in normal individuals during wakefulness or cognitive tasks (Bolduc et al., 2003). However, studies on hemispheric lateralization during NREM in primary insomnias (PIs) were relatively rare.

In this study, the power spectral analysis on PIs and good sleeper controls (GSCs) was conducted in six brain areas (frontal, midline, and occipital areas on both the left and right sides) during NREM sleep. The results could provide a deeper understanding of the biological mechanisms of insomnia. It would present valuable insights in future studies on the analysis of EEG power of insomnia in electrode selection.

## 2 Methods

### 2.1 Participants

The Sleep Center of the First People’s Hospital of Yunnan Province conducted this cohort study from July 2023 to February 2024. This study included 48 PIs and 30 age-and sex-matched GSCs. PIs and GSCs were selected from men and non-pregnant women aged 18–60. PIs were recruited from outpatient clinics at the First People’s Hospital of Yunnan Province, while GSCs were recruited through word-of-mouth referrals. After signing informed consent forms, all participants underwent questionnaire assessments and PSG examinations (data collection of over 8 h) at the Sleep Center of the First People’s Hospital of Yunnan Province.

The inclusion criteria of PIs was: clinical diagnosis of PIs meeting ICSD-3 diagnostic criteria. The exclusion criteria for PIs were: (1) any other sleep disorders, such as periodic limb movements and abnormalities in circadian rhythms; (2) any secondary insomnia, such as insomnia coinciding with menopause in women aged over 45 years; (3) Apnea-hypopnea index (AHI) > 15 and periodic limb movement arousal index (PMLSI) > 15.

The typical exclusion criteria for all participants were: (1) any severe cardiorespiratory diseases, brain diseases, immune system diseases, pain, thyroid dysfunction, metabolic diseases, and other severe or unstable health conditions; (2) mental illness: neither group meets the criteria for anxiety or depression diagnosis. PIs have any significant history of mental illness in the past 6 months, and GSCs have a history of current or past insomnia or any major mental disorder; (3) any substances or drugs that affect sleep, including antipsychotic medications, smoking, coffee consumption of 4 cups per 24 h and alcohol consumption of 14 drinks per week.

### 2.2 Scale assessments

The questionnaire assessments were conducted by a neurologist and a psychiatrist, both professionally trained. The inter-rater reliability was set at 90%. If the consistency falls below 90%, any discrepancies were resolved by a third rater.

The Pittsburgh Sleep Quality Index (PSQI) is one of the most widely used tools for assessing sleep quality globally. A score between 8 and 14 indicates mild insomnia, 14–21 indicates moderate insomnia, and ≥ 21 indicates severe insomnia. The Hamilton Anxiety Scale (HAMA) reflects the severity of anxiety symptoms. A score < 7 indicates no anxiety, and a score ≥ 14 indicates definite anxiety. The Hamilton Depression Scale (HAMD) is the most commonly used scale to assess depressive states. A score < 7 indicates normal, and a score ≥ 17 indicates depression. The Ford Insomnia Response to Stress Test (FIRST) evaluates an individual’s insomnia reactivity to the environment, with a total score ranging from 16 to 80. A score ≥ 18 can predict the onset of new insomnia.

### 2.3 Polysomnography

The participants were instructed to arrive at the sleep center around 8: 00 PM each night for PSG examination. Before coming to the sleep center, participants were asked to avoid alcohol, drugs, excessive caffeine, and nicotine. Bedtime and lights-out time were determined based on the participants’ self-reported bedtime over the past 2 weeks. PSG recording lasted for no less than 8 h.

PSG was conducted using an ambulatory Somte PSG (Neurotrace Mukie PSM-A, China). The sampling rate was 512 Hz, and the filter settings were as follows: high pass filter 0.1 Hz; low pass filter 50 Hz; notch frequency 60 Hz. Surface electrodes recorded included six EEG (F4/M1, C4/M1, O2/M1, F3/M2, C3/M2 and O1/M2), two electrooculogram (EOG), submental electromyography (EMG), electrocardiogram (ECG) and two reference electrodes (M1and M2). Other electrodes included a thermistor, nasal cannula, snoring sensor, thoracic and abdominal belt, and oxygen saturation probe. All recordings were acquired according to the American Academy of Sleep Medicine scoring rules guideline (Rundo and Downey III, 2019). The same experienced, registered PSG technologist scored all sleep recordings blind to the subject diagnostic group using the revised AASM 2.2 sleep scoring criteria.

### 2.4 Spectral analysis

Sleep staging was performed using sleep analysis software, with EEG data segmented into 30 s before and after sleep stage transitions. The analysis excluded artifacts containing body movements, respiration, leg movements, and other awakenings. The artifact-free EEG data during all NREM stages (i.e., N1, N2 and N3) were exported in European Data Format (EDF) and imported into MATLAB software (Version 2022b, Mathworks, United States) for spectral analysis.

Using the EEGLAB toolbox and ERPLAB in MATLAB (Math works, R2023b), raw data were imported at a sampling rate of 512 Hz and down-sampled to 256 Hz. The linked ear electrodes were used as a reference and segmented the 30-s NREM sleep epochs into 4-s (4-s) mini-epochs (Buysse et al., 2008; Kang et al., 2018). Semi-automatic artifact rejection procedures were used to remove channels and epochs with high-amplitude noise (Liu et al., 2021). Using the absolute threshold method, trials were selected to exclude those exceeding ±80 μV for each channel. Power spectrum in these ranges across all 4-s NREM epochs for each channel were plotted and visually inspected. When epochs showed significantly higher or lower amplitude power without exceeding the automatic threshold, we lowered the threshold and removed the exceeding epochs. Channels with artifacts affecting a majority of the recording were removed. We utilized additional spectral-based and topographic procedures to eliminate individual channels exhibiting markedly higher power than adjacent channels.

Selected 4-s NREM epochs in each EEG electrode were Hanning-tapered and Fourier-transformed by the FFT algorithm (with 50% overlapping windows) to obtain the power spectrum density at each frequency. We assessed the following seven frequency bands: delta (1–4 Hz), theta (4–8 Hz), alpha (8–12 Hz), sigma (12–16 Hz), beta1 (16–24 Hz), beta2 (24–32 Hz), and gamma (32–50 Hz), with each band inclusive of the lower boundary but not the upper boundary.

### 2.5 Data analysis

SPSS (version 17.0, SPSS, Inc., Chicago, IL, United States) was used for statistical analyses. The data were presented as mean ± standard deviation if they are normally distributed (or approximately normally distributed). If they are not, the data were presented as the median (with interquartile range in brackets). Histograms, q-q plots, and Shapiro–Wilk’s tests assessed the data normality. Normally distributed variables were compared using a one-way analysis of variance or T-test, and the non-normally distributed variables using the Mann–Whitney U test. The Levene test was used to test variance homogeneity. The one-way testing was performed on data with homogeneity of variances and multiple *post-hoc* comparisons between the six areas using the Scheffe method. Welch’s test for correction was applied for data with heterogeneity of variances, and Tamhane T2 was utilized for *post-hoc* multiple comparisons. Categorical variables were presented as the frequency distribution and compared using the chi-squared or Fisher’s exact test.

The adjusted *p*-values were calculated for multiple comparisons using the Least Significance Difference method. A false discovery rate of *p* < 0.05 was considered statistically significant.

## 3 Results

### 3.1 Group characteristics

The demographic and clinical characteristics were shown in Table 1. PIs and GSCs differed on the HAMA, HAMD, FIRST and PSQI. The HAMD and HAMA scores of PIs and GSCs fell within the normal range, but the HAMD and HAMA scores of PIs were higher than those of GSCs (*p* < 0.01). Notably, the median PSQI score for PIs was 16, falling within the range of moderate insomnia. The difference between the two groups was highly significant (*p* < 0.01). There were no significant differences in age, sex, height, weight, and race between groups.

**Table 1:** Demographics and scale scores of PIs and GSCs.

	PIs *n* = 48	GSCs *n* = 30	*p*-value
Age	37(32–52)	37(32–49)	0.90
Sex (Female)	32/48 (66.67%)	19/30 (63.33%)	0.83
Education (Y)	15 (8–15)	11 (8–18)	0.08
BMI	21 (20–24)	22 (20–24)	0.43
HAMA	7 (4–11)	1 (0–3)	<0.01**
HAMD	7 (4–10)	1 (0–2)	<0.01**
PSQI	16 (13–18)	1 (0–3)	<0.01**
FIRST	18 (17–22)	11 (9–14)	<0.01**

Data were presented as count (percentage) and median (interquartile range). HAMA, Hamilton Anxiety Scale; HAMD, Hamilton Depression Scale; PSQI, Pittsburgh Sleep Quality Index; FIRST, Ford insomnia response to stress test. **p < 0.01.

### 3.2 Group differences on sleep diary and PSG

As shown in Table 2, PIs and GSCs differed in most subjective sleep measures. PIs had more than 1 h of subjective sleep latency (SOL) and total sleep duration (TST) of less than 5 h. The differences between subjective and objective measures showed that PIs significantly overestimated sleep latency and underestimated total sleep duration and efficiency compared to GSCs.

**Table 2:** Sleep diary and polysomnography (PSG) characteristics of PIs and GSCs.

	PIs	GSCs	Homogeneity of variance	t or Z score	*p*-value
	*n* = 48	*n* = 30			
Sleep diary					
TIB(min)	492.29 (67.99)	497.3 (46.97)	0.058	−0.356	0.723
SOL (min)	78.02 (43.11)	16.50 (13.67)	0	−6.88	<0.001***
TST(min)	295.00 (70.11)	452.6 (43.15)	0.004	−7.074	<0.001***
SEI (%)	60.11 (13.17)	91.22 (6.06)	0	−7.215	<0.001***
PSG					
TIB(min)	484.34 (52.46)	495.2 (64.75)	0.25	−0.814	0.418
SOL(min)	39.78 (42.76)	12.07 (14.21)	0.001	−4.182	<0.001***
WASO(min)	84.46 (54.83)	39.40 (30.34)	0.001	−3.765	<0.001***
TWT(min)	123.51 (83.58)	51.47 (37.10)	0	−4.319	<0.001***
WT	73.48 (69.76)	24.70 (26.96)	0.005	−4.32	<0.001***
TST(min)	361.70 (80.68)	438.0 (58.46)	0.038	−4.021	<0.001***
SEI (%)	74.64 (15.91)	88.74 (7.24)	0	−4.514	<0.001***
N1 (%)	10.25 (6.75)	7.11 (3.42)	0.002	−1.736	0.083
N2 (%)	49.24 (16.11)	47.58 (7.18)	0	0.118	0.906
N3 (%)	6.61 (5.51)	14.96 (5.18)	0.364	−6.656	<0.001***
REM (%)	12.11 (5.92)	16.99 (6.36)	0.902	−3.442	<0.01**

Data was presented as mean (SD). TIB, time in bed; SOL, sleep onset latency; WASO, wakefulness after sleep onset; TWT, total wake time; WT, wake times; TST, total sleep time = TIB − TWT; SEI, sleep efficiency index = TST/TIB; REMS, rapid eye movement sleep; non-REMS (NREMS) stages N1, N2 and N3 (slow wave sleep). If Levene’s variance test *p*-value > 0.05, a *t*-test (t-score) was used; if *p*-value < 0.05, a Mann–Whitney U test (Z-score) was used. *p < 0.05; **p < 0.01; ***p < 0.001.

Objective PSG measures of sleep efficiency (SEI), TST, and SOL differed between the two groups (*p* < 0.01). PIs had poor sleep continuity, as evidenced by many awakenings after sleep onset (WASO; 84.46 ± 54.83; 39.40 ± 30.34, respectively, *p* < 0.001) and a long total awakening duration (TWT; 123.51 ± 83.58; 51.47 ± 37.10, respectively, *p* < 0.001). PIs had a lighter sleep, with a higher percentage of N1 (10.25 ± 6.75; 7.11 ± 3.42, respectively, *p* = 0.008) and a significant reduction in the more profound sleep period, i.e., the N3 period of sleep (6.61 ± 5.51; 14.96 ± 5.18, respectively, *p* < 0.001), and a decrease in the ratio of REM (12.11 ± 5.92%; 16.99 ± 6.36, respectively, *p* < 0.001) in PIs compared to GSCs.

### 3.3 Differences in the absolute power spectrum between GSCs and PIs in different areas

**Figure 1:**
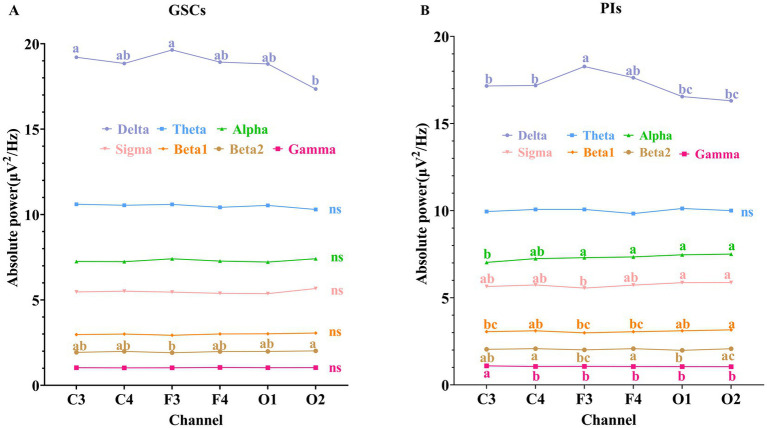
Comparison of absolute power spectrum in different areas during NREM sleep. **(A)** GSCs; **(B)** PIs. The labeling indicated *post-hoc* differences between groups at the *p* < 0.05. “a” represented the largest mean value, and “b,” “c,” “d” were sorted in descending order of the mean values. Groups without the same letter indicated *post-hoc* differences between groups at *p* < 0.01.

As shown in Figure 1 and Table 3. In GSCs, apart from delta power and beta2 power showing differences across various areas, no significant differences were observed in other frequency band’s power between different areas. The delta power of GSCs was highest in F3 and C3.

**Table 3:** Differences in the power spectrum between GSCs and PIs in different areas.

	C3	C4	F3	F4	O1	O2	*Post-hoc* test *p*-values	*p*-value
GSCs								
Delta	19.21 (1.88)^a^	18.85 (1.72)^ab^	19.64 (2.00)^a^	18.93 (1.85)^ab^	18.82 (2.12)^ab^	17.35 (2.08)^b^	0.729	0.0000****
Theta	10.60 (0.83)	10.54 (0.64)	10.60 (0.67)	10.42 (0.86)	10.53 (0.71)	10.30 (1.01)	0.399	0.667
Alpha	7.25 (0.46)	7.24 (0.31)	7.41 (0.36)	7.28 (0.47)	7.22 (0.28)	7.41 (0.44)	0.208	0.217
Sigma	5.67 (0.56)	5.51 (0.48)	5.46 (0.53)	5.39 (0.38)	5.37 (0.52)	5.57 (0.56)	0.34	0.223
Beta1	2.97 (0.18)	3.00 (0.14)	2.93 (0.17)	3.01 (0.21)	3.02 (0.19)	3.06 (0.17)	0.097	0.14
Beta2	1.93 (0.10)^ab^	1.99 (0.12)^ab^	1.91 (0.09)^b^	1.98 (0.14)^ab^	1.99 (0.09)^ab^	2.02 (0.18)^a^	0.056	0.01**
Gamma	1.03 (0.03)	1.03 (0.01)	1.03 (0.02)	1.05 (0.04)	1.03 (0.02)	1.04 (0.04)	0.003	0.133
PIs								
Delta	17.16 (1.53)^b^	17.18 (1.36)^b^	18.27 (1.29)^a^	17.63 (1.29)^ab^	16.54 (1.29)^bc^	16.31 (1.03)^bc^	0.4823	0.0000****
Theta	9.95 (0.50)	10.07 (0.42)	10.07 (0.58)	9.83 (0.55)	10.12 (0.58)	10.00 (0.39)	0.0785	0.0622
Alpha	7.03 (0.41)^b^	7.25 (0.39)^ab^	7.30 (0.32)^a^	7.34 (0.37)^a^	7.47 (0.59)^a^	7.50 (0.45)^a^	0	0.0000****
Sigma	5.65 (0.35)^ab^	5.74 (0.35)^ab^	5.56 (0.28)^b^	5.72 (0.38)^ab^	5.87 (0.33)^a^	5.88 (0.33)^a^	0.6352	0.0000****
Beta1	3.05 (0.17)^bc^	3.11 (0.18)^ab^	2.99 (0.13)^bc^	3.05 (0.11)^bc^	3.10 (0.16)^ab^	3.16 (0.15)^a^	0.0006	0.0000****
Beta2	2.04 (0.08)^ab^	2.08 (0.09)^a^	2.01 (0.08)^bc^	2.08 (0.11)^a^	1.98 (0.07)^b^	2.07 (0.11)^ac^	0.0297	0.0000****
Gamma	1.09 (0.05)^a^	1.06 (0.03)^b^	1.06 (0.04)^b^	1.05 (0.03)^b^	1.05 (0.05)^b^	1.04 (0.03)^b^	0	0.0000****

Data was presented as mean (SD). *Post-hoc* test *p*-values ≥ 0.05 indicated equal variance (ANOVA); *post-hoc* test *p*-values < 0.05 indicated unequal variance (Welch’s test). *p*-values were from one-way ANOVA or Welch’s test. “a” represented the largest mean value, and “b,” “c,” “d” were sort in descending order of the mean values. Groups without the same letter indicated *post-hoc* differences between groups at *p* < 0.01. *p < 0.05; **p < 0.01; ***p < 0.001; ****p < 0.0001.

In PIs, the delta power was also highest in F3. The beta2 power was the highest in C4 and F4, beta1 power in C4 and O2, gamma power in C3, and alpha and sigma powers in O1 and O2. The theta, alpha, sigma, beta1, and gamma power did not show significant differences in different areas in PIs.

### 3.4 Power spectral comparison between PIs and GSCs in different brain areas

**Figure 2:**
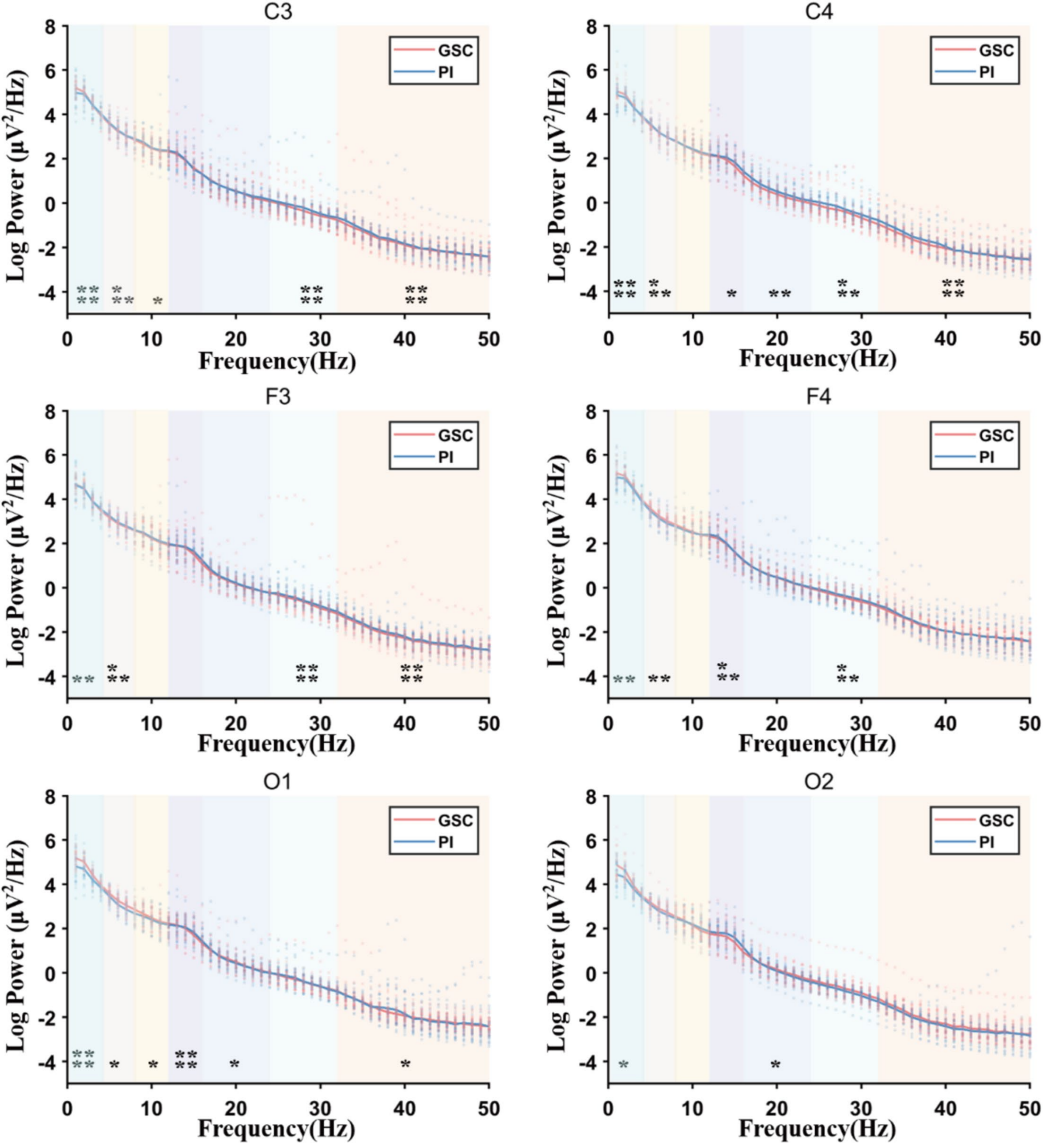
The full power spectral density (PSD) plots comparing Primary Insomnia (PIs) and Good Sleep Controls (GSCs). Delta (1–4 Hz), theta (4–8 Hz), alpha (8–12 Hz), sigma (12–16 Hz), beta1 (16–24 Hz), beta2 (24–32 Hz), and gamma (32–50 Hz); Sigma power increased mainly in F4 and gamma power increased mainly in C3, F3 and C4. *p < 0.05; **p < 0.01; ***p < 0.001; ****p < 0.0001.

**Figure 3:**
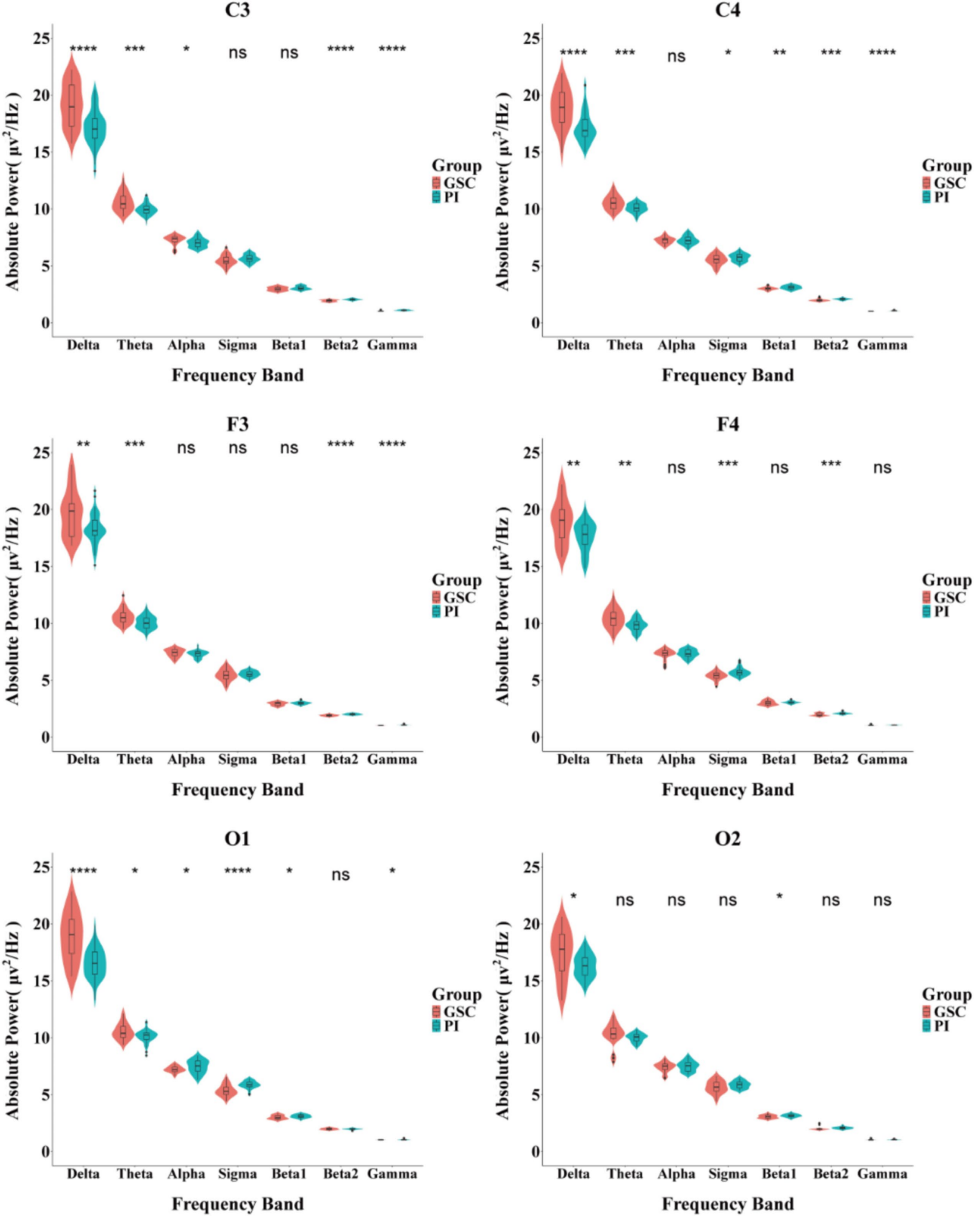
Comparison of the absolute power spectrum between PIs and GSCs in different brain areas during NREM sleep. *p < 0.05; **p < 0.01;***p < 0.001; ****p < 0.0001.

As shown in Figures 2, 3 and Table 4 and Supplementary Image 1. The difference in delta power between the two groups was statistically significant in all six areas. The difference in theta power between the two groups was statistically significant in C4, F3, F4, O1 and O2. The alpha power of PIs was lower than GSCs in C3 and higher in O1. The sigma power of PIs was significantly higher than GSCs in F4 and C4. The beta1 power of PIs was substantially higher in C4. The beta2 power of PIs was considerably higher in C4, F4, C3, and F3; meanwhile, C4 and F4 had the highest power in all six areas. The gamma power of PIs was significantly higher than that of GSCs in C3, F3 and C4, and the gamma power of PIs was highest in C3.

**Table 4:** Power spectral comparison between PIs and GSCs in different brain areas.

	PIs	GSCs	Homogeneity of variance	*t*-score	*p*-value
	*n* = 48	*n* = 30			
Delta					
C3	17.16 (1.53)	19.21 (1.88)	0.0944	−5.28	0.0000****
C4	17.18 (1.36)	18.85 (1.72)	0.0989	−4.752	0.0000****
F3	18.27 (1.29)	19.64 (2.00)	0.0031	11.111	0.0017**
F4	17.63 (1.29)	18.93 (1.85)	0.0378	11.346	0.0015**
O1	16.54 (1.29)	18.82 (2.12)	0.0007	28.037	0.0000****
O2	16.31 (1.03)	17.35 (2.08)	0	6.589	0.0143*
Theta					
C3	9.95(0.50)	10.6 (0.83)	0.0007	15.175	0.0003****
C4	10.07 (0.42)	10.54 (0.64)	0.0139	12.716	0.0009****
F3	10.07 (0.58)	10.60(0.67)	0.5709	−3.68	0.0004****
F4	9.83(0.55)	10.42 (0.86)	0.0079	11.371	0.0016**
O1	10.12 (0.58)	10.53 (0.71)	0.1401	−2.789	0.01*
O2	10 (0.39)	10.3 (1.01)	0.0005	2.417	0.1292
Alpha					
C3	7.03 (0.41)	7.25 (0.46)	0.9008	−2.238	0.0282*
C4	7.25 (0.39)	7.24 (0.31)	0.1903	0.055	0.9565
F3	7.30 (0.32)	7.41 (0.36)	0.2368	−1.415	0.1614
F4	7.34 (0.37)	7.28 (0.47)	0.5184	0.655	0.5147
O1	7.47 (0.59)	7.22 (0.28)	0	6.022	0.0165*
O2	7.50 (0.45)	7.42 (0.44)	0.6174	0.747	0.1292
Sigma					
C3	5.65 (0.35)	5.47 (0.49)	0.0533	1.851	0.068
C4	5.74 (0.35)	5.51 (0.48)	0.1039	2.362	0.0207*
F3	5.56 (0.28)	5.46 (0.53)	0.0009	0.821	0.3703
F4	5.73 (0.38)	5.39 (0.38)	0.8969	3.781	0.0003***
O1	5.87 (0.33)	5.37 (0.52)	0.011	22.169	0.0000***
O2	5.88 (0.33)	5.67 (0.56)	0.0015	3.349	0.0744
Beta 1					
C3	3.05 (0.17)	2.97 (0.18)	0.5123	1.929	0.0575
C4	3.12 (0.18)	3.00 (0.14)	0.0533	2.707	0.0084**
F3	2.99 (0.13)	2.94 (0.17)	0.0252	2.1	0.1537
F4	3.05 (0.11)	3.01 (0.21)	0	0.982	0.3278
O1	3.11 (0.16)	3.02 (0.19)	0.1457	2.189	0.0317*
O2	3.16 (0.15)	3.06 (0.17)	0.1644	2.636	0.0102*
Beta 2					
C3	2.04 (0.08)	1.94 (0.10)	0.0296	23.602	0.0000****
C4	2.08 (0.09)	1.99 (0.12)	0.1641	4.05	0.0001****
F3	2.01 (0.08)	1.91 (0.09)	0.3313	5.139	0.0000****
F4	2.08 (0.09)	1.98 (0.14)	0.0003	12.759	0.0009****
O1	1.98 (0.07)	1.99 (0.09)	0.011	0.033	0.8563
O2	2.07 (0.11)	2.02 (0.18)	0.1696	1.652	0.1027
Gamma					
C3	1.09 (0.05)	1.03 (0.03)	0.0004	37.927	0.0000****
C4	1.06 (0.03)	1.03 (0.01)	0.0171	47.618	0.0000****
F3	1.06 (0.04)	1.03 (0.02)	0.0193	23.602	0.0000****
F4	1.05 (0.03)	1.05 (0.04)	0.3176	1.062	0.2916
O1	1.05 (0.05)	1.03 (0.02)	0.0012	4.446	0.0390*
O2	1.04 (0.03)	1.04 (0.04)	0.0936	0.836	0.4057

Data was presented as mean (SD). Homogeneity of variance check: *p* ≥ 0.05 indicated equal variance (ANOVA); *p* < 0.05 indicated unequal variance (Welch’s test). *p*-values were from one-way ANOVA or Welch’s test. *p < 0.05; **p < 0.01; ***p < 0.001; ****p < 0.0001.

### 3.5 The correlation analysis of different parameters in PIs

**Figure 4:**
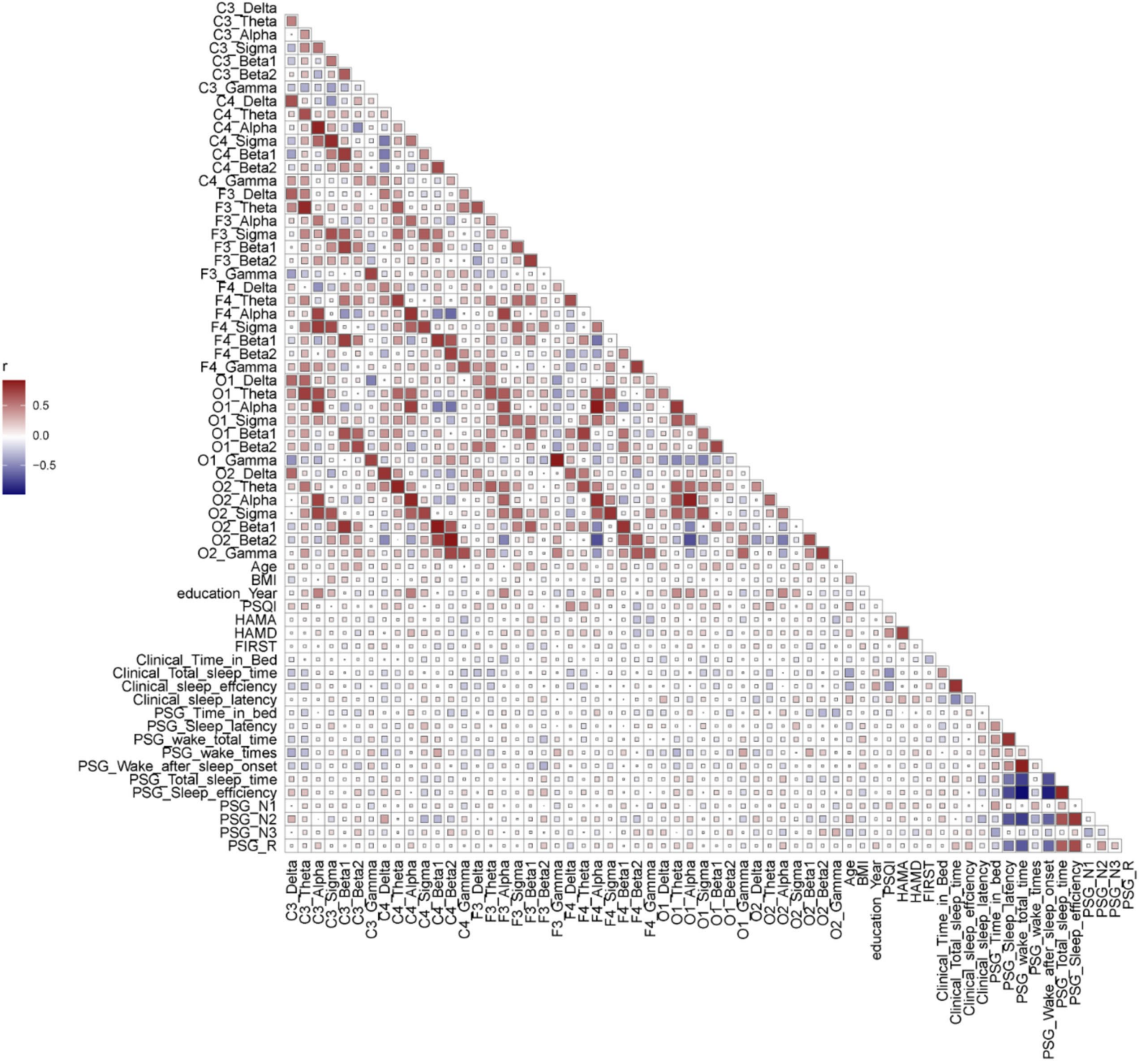
The correlation analysis of parameters in PI patients (baseline, scales, supervisor sleep, PSG parameters, and power spectra of different channels). Spearman correlation analysis was performed between the parameters. Red represented a positive correlation, while blue represented a negative correlation. The larger the r value, the deeper the color. The size of squares represented the *p*-value.

As shown in Figure 4 and Supplementary Table 1. In PIs, alpha power was positively correlated with education years across six areas. Beta1 power was positively correlated with age in F3 (*r* = 0.3283, *p* = 0.02) and the number of awakenings in C4 (*r* = 0.3291, *p* = 0.02) and O2 (*r* = 0.3018, *p* = 0.04). Beta2 power was positively correlated with age in C3 (*r* = 0.3297, *p* = 0.02) and negatively with WASO in F3 (*r* = −0.3344, *p* = 0.02).

## 4 Discussion

In this study, PIs had lower delta power and higher beta1, beta2, and gamma power than GSCs, suggesting reduced deep sleep and increased arousal levels. During NREM, GSCs show no significant disruption in the power of brain activity between the two hemispheres, whereas PIs display significant disruptions. No significant correlations were found between HAMA, HAMD, PSG parameters and power spectra across different areas. The beta1 power was positively correlated the number of awakenings.

PIs exhibited an increased beta power during NREM sleep or at sleep onset (Neu et al., 2015; Kay et al., 2019), indicating potential over-alertness or rumination at sleep onset or during sleep (Freedman, 1986; Buysse et al., 2008; Cervena et al., 2014). However, some studies did not observe a similar increasing beta power during NREM sleep in PIs (Bastien et al., 2003). The different conclusions underlying the different methods were drawn due to the different areas and frequency bands. The power spectral analysis in different areas and frequency bands is significant. This study marked the first specialized comparison of power spectral analysis during NREM sleep in different areas and across different frequency bands between PIs and GSCs.

### 4.1 In PIs, beta2 power increased in all areas, while sigma power increased on the right side

In GSCs, reduced delta power in the right hemisphere and increased beta2 power in the right hemisphere suggested heightened alertness (Riemann et al., 2010; Cabrera et al., 2024) during the first night of wearing PSG. However, these changes in alertness did not reach the threshold for awakening, leading to fewer awakening events. In contrast, PIs showed a more pronounced response to environmental adaptation (higher FIRST scores than GSCs; Van Someren, 2021), characterized by significant increases in alertness, particularly in beta 2 power, which was elevated across both hemispheres but more so in the right hemisphere. The right hemisphere exhibited higher sensitivity to alertness during the awakening process (Swarnkar et al., 2007). This was accompanied by an increase in awakening events, with WASO, TWT and WT significantly increased in PIs.

However, no strong correlation between beta2 power and increased alertness was observed in PIs. This might be due to the exclusion of artifacts (e.g., movement artifacts associated with awakenings) during data analysis, which could have impacted the detection of the relationship between beta2 power and awakenings. Future studies incorporating additional alertness indicators, such as cortisol levels and heart rate variability (HRV), along with more artifact-free stereotactic-electroencephalography (SEEG) data, might provide further insights into this phenomenon.

Most previous power spectral analyses on insomnia did not subdivide the beta band into beta1 and beta2. It could not provide a better understanding of the biological significance of EEG (Van Albada and Robinson, 2013).

The sigma power increased on the right side in PIs, which aligned with a hypothesis that sigma power elevation was linked to the activation of emotional memory traces, predominantly in the right hemisphere. According to the hyperarousal theory of insomnia, insomnias require substantial encoding and transformation of emotions during NREM sleep. Amygdala adaptation necessitates stable NA pauses during REM sleep and the reactivation of memory traces during the pre-REM period, indicated by an increase in sleep spindles (sigma) (Sterpenich et al., 2007; Van Der Helm et al., 2011). Hence, alterations in sigma power in insomnias are expected (Spiegelhalder et al., 2012). At the same time, the emotional rhythms prefer to be processed in the right hemisphere (Ahern and Schwartz, 1985).

The clinical treatment strategy of repetitive transcranial magnetic stimulation (rTMS) and transcranial direct current stimulation (tDCS) for insomnia followed the protocol of high-frequency stimulation of the left hemisphere and low-frequency inhibition of the right hemisphere (Perera et al., 2016).

### 4.2 The gamma power of PIs significantly increased on the left side

The left side would be a dominant brain area for declarative memory consolidation. The increased gamma power during NREM sleep in PIs could be an active declarative memory consolidation process.

Usually, the gamma oscillation during wakefulness was associated with memory encoding and retrieval. Using deep electrodes in conjunction with scalp electrodes had found spontaneous gamma oscillations, particularly in the depolarized state of slow-wave oscillations (up states) during NREM sleep (Mölle et al., 2009; Valderrama et al., 2012). The local field potentials (LFPs) detected the gamma oscillation in sleeping or anesthetized animals (Mukovski et al., 2007; Mena-Segovia et al., 2008). During NREM sleep, gamma oscillations are associated with memory consolidation, transforming initially unstable memories from wakefulness into more stable representations (Diekelmann and Born, 2010).

Increased gamma power during NREM sleep was detected using the bilateral average montage (Perlis et al., 2001a; Perlis et al., 2001b; Buysse et al., 2008). However, they did not clarify the biological significance. The increase in gamma power in different brain areas would reveal important physiological significance.

### 4.3 Beta1 power positively correlates with number of awakenings on the right side

The positive correlation between beta1 power and the number of awakenings suggested that individuals exhibiting higher beta1 activity during sleep were more prone to sleep fragmentation. This relationship implied that elevated beta1 power might serve as a biomarker for sleep maintenance difficulties, where increased cortical arousal interrupted sleep, leading to frequent awakenings. Such disruptions could degrade sleep quality and contribute to the subjective experience of non-restorative sleep commonly reported in insomnia (Shi et al., 2022; Hermans et al., 2021). Additionally, awakenings were often characterized by asynchronous information processing between the left and right hemispheres of the brain (Swarnkar et al., 2006; Swarnkar et al., 2007), with the right hemisphere typically showing greater sensitivity to arousal (Liotti and Tucker, 1992).

A limitation of this study was that only six brain areas were compared, which might not provide a comprehensive understanding of the biological mechanisms of insomnia. Future studies using standard PSG combined with high-density EEG (hdEEG) for synchronous recordings in PIs could address this limitation. Comparing the power spectra across different stages of sleep (REM, NREM and wakefulness) would offer more detailed insights.

## 5 Conclusion

This study marked the first specialized comparison of power spectral analysis during NREM sleep in different areas and across different frequency bands. PIs showed increased beta2, sigma and gamma power compared to GSCs. During NREM, PIs showed significant disruption in the power of brain activity between the two hemispheres. Increased gamma power was predominantly found on the left side. Sigma power increased dramatically on the right side. Beta1 Power positively correlates with number of awakenings on the right side. These findings revealed the hemispheric lateralization characteristics of power spectral disturbances during NREM in PIs and provided valuable insights for selecting electrode placements in future power spectral analyses of PIs.

## Data Availability

The datasets presented in this study can be found in online repositories. The names of the repository/repositories and accession number (s) can be found in the article/Supplementary material.
